# Reversible post–head-shaking nystagmus in horizontal canal BPPV as a mechanical mimic of vestibular hypofunction: a case report

**DOI:** 10.1007/s00405-026-10640-y

**Published:** 2026-10-13

**Authors:** Hadas Ben-Rubi Shimron, Andrea Castellucci, Yoav Gimmon

**Affiliations:** 1 Department of Otolaryngology- Head and Neck Surgery, Sheba Medical Center, Tel-Hashomer, Ramat Gan, Israel; 2 Department of Physical Therapy, Faculty of Social Welfare & Health Sciences, University of Haifa, Haifa, Israel; 3 Department of Surgery, ENT Unit, Azienda USL – IRCCS di Reggio Emilia, Reggio Emilia, Italy

**Keywords:** BPPV, Horizontal semicircular canal, Post-head-shaking nystagmus, Pseudo-spontaneous nystagmus, Differential diagnosis, Case report

## Abstract

**Purpose:**

To describe transient post–head-shaking nystagmus associated with horizontal canal benign paroxysmal positional vertigo (BPPV) and its resolution following canalith repositioning.

**Methods:**

A 78-year-old woman presented with positional vertigo. Video-oculography revealed left-beating pseudo-spontaneous nystagmus and a robust left-beating pHSN, initially suggestive of uncompensated unilateral vestibulopathy. Further bedside assessment, including the Head Pitch test and Supine Roll test, demonstrated direction-changing horizontal nystagmus consistent with left horizontal canalolithiasis.

**Results:**

After an unsuccessful Lempert maneuver, a Gufoni maneuver was performed, resulting in immediate resolution of vertigo and nystagmus. At 10-day follow-up, no spontaneous, positional, or post-head-shaking nystagmus was observed. The patient remained asymptomatic throughout 14-month follow-up, with preserved horizontal vHIT responses at reassessment.

**Conclusion:**

This case suggests that post–head-shaking nystagmus in horizontal canal BPPV may arise from transient mechanically triggered asymmetry rather than a fixed vestibular deficit. Reliance on pHSN alone may lead to misdiagnosis and inappropriate diagnostic interpretation. Incorporating positional testing and reassessment following canalith repositioning may help avoid misinterpretation of post–head-shaking nystagmus as unilateral vestibular hypofunction.

**Supplementary information:**

The online version contains supplementary material available at https://doi.org/10.1007/s00405-026-10640-y.

## Introduction

The Head-Shaking Test (HST) is a commonly used bedside tool for detecting latent vestibular asymmetry in patients presenting with vertigo and dizziness [1]. The test involves oscillating the head in the horizontal plane for approximately 20s and observing for post-head-shaking nystagmus (pHSN), which may indicate uncompensated vestibular imbalance [1].

In unilateral vestibular hypofunction (UVH), pHSN is typically explained by the velocity storage mechanism, which temporarily retains asymmetrical neural input during head oscillation and releases it when movement stops. This results in pHSN following Ewald’s laws, usually beating toward the side with stronger input [2]. Atypical presentations are generally attributed to central pathologies and require neurological evaluation [3]. However, pHSN is not specific to vestibular hypofunction and has also been reported in BPPV and other vestibular disorders [2–7].

From a clinical perspective, reliance on pHSN as an isolated finding may therefore lead to diagnostic error. The HST should be interpreted within the context of a comprehensive vestibular examination, particularly when distinguishing between vestibular hypofunction and mechanically triggered disorders such as BPPV.

Although not typically used to diagnose BPPV, pHSN is reported in up to 48% of BPPV patients [4]. Lee and Kim [4] observed pHSN in 48% of horizontal canal BPPV (hc-BPPV) cases, including geotropic and apogeotropic forms. Similarly, Kim et al. [5] noted that 22.2% of patients with pHSN were eventually diagnosed with BPPV. Conversely, Lopez-Escamez et al. [6] found no significant difference in pHSN prevalence between hc-BPPV patients (24%) and healthy controls (29%), though nystagmus velocity was higher in the BPPV group. In all cases, no consistent pattern emerged, as nystagmus beat either toward or away from the affected side. Across these studies, findings were generally reported at a single time point without direct documentation of their evolution after canalith repositioning. Thus, the physiological significance of pHSN in BPPV remains uncertain.

Accurate differentiation between fixed vestibular deficits and mechanically triggered disorders such as BPPV is essential, because similar clinical findings may arise from different mechanisms and require different therapeutic approaches. While vestibular hypofunction is typically managed with gaze stabilization and adaptation exercises, BPPV requires canalith repositioning maneuvers (CRM). Misinterpretation of clinical signs may therefore lead to inappropriate treatment and delayed symptom resolution.

We present a longitudinal case of a patient with spontaneous nystagmus, pHSN, and concurrent BPPV. By tracking findings before and after treatment, we observed how nystagmus patterns evolved, supporting a transient mechanically triggered interpretation of the findings.

## Case description

A 78-year-old woman with recurrent BPPV and no history of orthopedic, neurologic, or head trauma, presented for reassessment due to persistent vertigo when rolling in bed. Two days prior, she underwent treatment for left posterior canal canalolithiasis at another clinic, reported as initially successful.

## Clinical examination

Oculomotor examination in room light revealed pseudo-spontaneous left-beating horizontal nystagmus, increasing on left gaze and decreasing on right gaze (Alexander’s law). With fixation removed via video goggles, the nystagmus persisted and intensified, maintaining the same gaze-dependent pattern (Online Resource 1).

The HST revealed robust horizontal, left-beating pHSN which decayed over 42 s (Online Resource 2). The Head Pitch Test (HPT), or Bow and Lean test, revealed direction-changing nystagmus: forward flexion enhanced the left-beating nystagmus, while backward extension induced right-beating nystagmus (Online Resource 3).

The left Dix-Hallpike test elicited intense left-beating horizontal nystagmus with vertigo. Testing to the right produced weaker right-beating nystagmus with mild vertigo. The Supine Head Roll Test (sHRT) revealed direction-changing paroxysmal geotropic horizontal nystagmus, more intense during left roll, consistent with left hc-BPPV (Online Resource 4).

## Intervention and follow-up

An initial Lempert (barbecue roll) maneuver failed to resolve symptoms. Subsequently, a Gufoni maneuver for left hc-BPPV was performed, leading to immediate resolution of vertigo on re-testing.

The patient reported complete relief immediately following treatment and sustained resolution at 10-day follow-up. Clinical reassessment revealed no spontaneous or pseudo-spontaneous nystagmus, a negative HST, and no nystagmus on HPT or sHRT (Online Resource 5).

During the subsequent year, the patient was contacted several times by telephone and consistently reported no dizziness or vertigo, with return to her previous level of function. At 14-month follow-up, she remained asymptomatic. Repeated clinical examination, including assessment for spontaneous nystagmus, HST, HPT, and sHRT, was negative. Horizontal Video Head Impulse testing (vHIT) demonstrated preserved vestibulo-ocular reflex responses bilaterally, with no reduced gain or corrective saccade pattern suggestive of unilateral horizontal canal hypofunction (Online Resource 6).

## Discussion

pHSN has previously been reported in patients with BPPV [4–6]; however, most prior studies have described its presence in cross-sectional cohorts without documenting its evolution following treatment. In contrast, the present case provides sequential documentation demonstrating that spontaneous nystagmus and pHSN resolved after successful CRM. This temporal relationship supports a mechanically triggered transient vestibular asymmetry rather than a persistent unilateral vestibular deficit.

Previous work has questioned the mechanisms underlying pHSN, with both central (velocity storage) and peripheral explanations proposed [2, 4, 6, 7]. Mechanical factors may be particularly relevant in BPPV. For example, Castellucci et al. [8] described a case of horizontal canal BPPV mimicking acute unilateral vestibulopathy, in which spontaneous nystagmus and apparent canal hypofunction were attributed to a canalith jam, highlighting that mechanically triggered pathology can produce findings typically associated with vestibular asymmetry.

In the present case, spontaneous left-beating nystagmus and prolonged pHSN could have suggested right-sided vestibular hypofunction. However, direction-changing nystagmus during the HPT and the resolution of all findings following CRM suggested a transient BPPV-related vestibular asymmetry rather than a persistent unilateral deficit. This temporal pattern raises the possibility that pHSN may arise from a mechanically triggered asymmetry in vestibular input rather than from a persistent imbalance.

The clinical course suggests a coherent mechanically triggered sequence. The patient had undergone treatment for left posterior canal canalolithiasis two days before the present examination, raising the possibility that posterior-to-horizontal canal conversion contributed to the presence of debris within the horizontal canal [9]. This possibility does not alter the diagnosis of left horizontal canal canalithiasis, which was supported by the direction-changing geotropic nystagmus on positional testing and by the resolution of findings after the Gufoni maneuver. Rather, it provides a plausible explanation for the origin of the mechanically active horizontal-canal debris. The present findings are consistent with pHSN arising from a transient vestibular asymetry related to horizontal canal BPPV rather than from persistent unilateral vestibular deficit. Head-shaking may have mobilized freely floating otoconial debris within the horizontal canal, generating asymmetric endolymphatic flow and unequal cupular deflection [10]. The 42-s decay of the observed pHSN suggests that the response was unlikely to reflect only a brief mechanically triggered event. Rather, the prolonged decay may reflect a sustained peripheral asymmetry generated by continued debris-related endolymphatic flow, potentially combined with central temporal integration of the asymmetric vestibular signal [11]. Whether velocity storage contributed to the observed 42-s decay cannot be established from the present case. This interpretation therefore remains one possible explanation for how horizontal canal BPPV may generate pHSN that mimics vestibular hypofunction.

The long-term follow-up further supports this interpretation. Although vHIT was not performed at the initial presentation, the patient remained asymptomatic over the following year, and the 14-month reassessment demonstrated preserved horizontal vHIT responses. These findings do not exclude a transient or compensated vestibular asymmetry at the time of presentation, but they argue against persistent fixed unilateral horizontal canal hypofunction as the primary explanation for the original pseudo-spontaneous and post-head-shaking nystagmus.

**Fig. 1:**
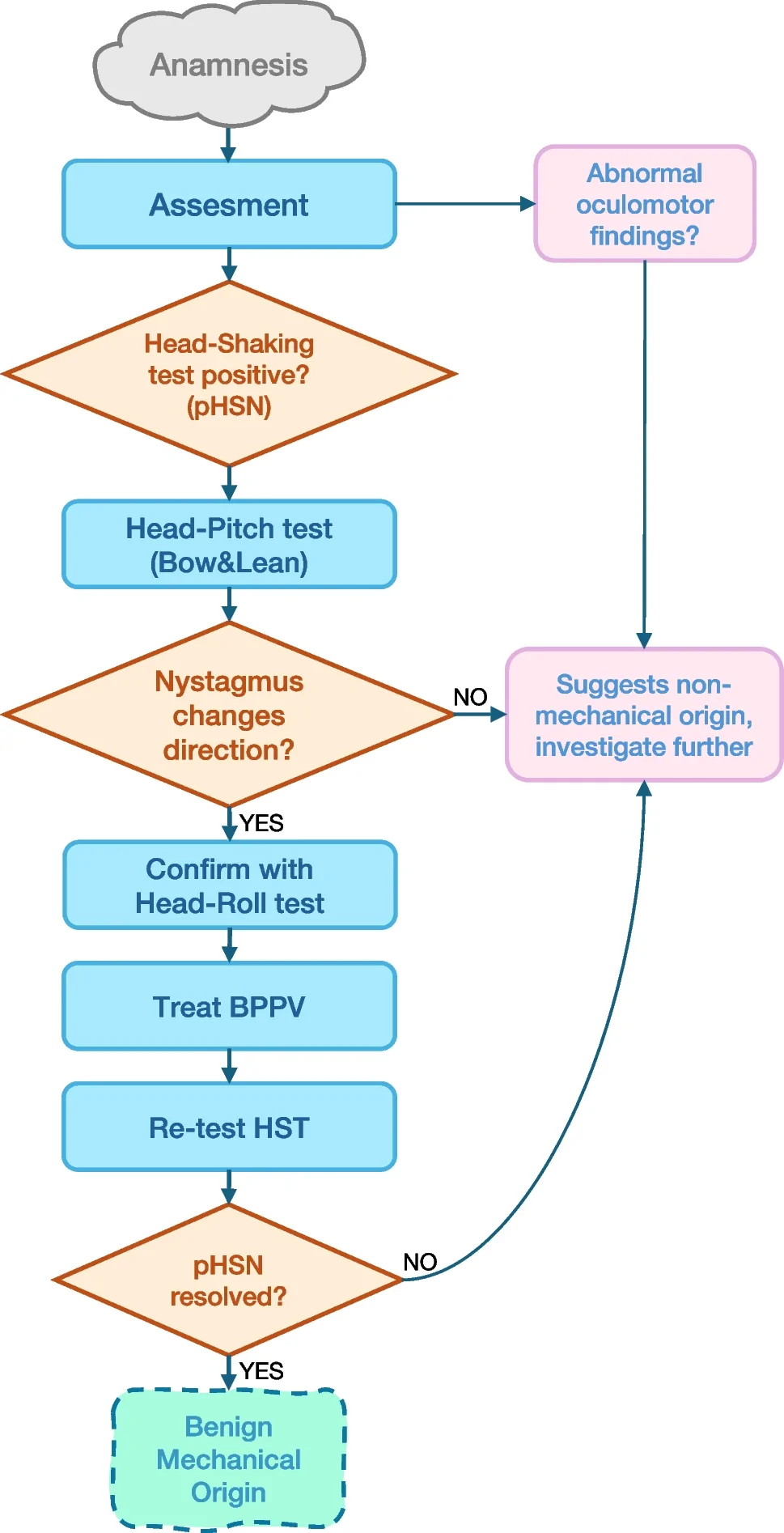
Interpretive framework for pHSN in suspected mechanically triggered vestibular pathology. The central vertical pathway (blue/orange) outlines the sequence for supporting a mechanically triggered etiology (BPPV), while the lateral branches (purple) indicate "exit points" where fixed or atypical findings require investigation for central or persistent neural pathology. Abbreviations: BPPV- Benign Paroxysmal Positional Vertigo; HST- Head Shaking Test; pHSN- post Head Shaking Nystagmus

From a clinical perspective, this case highlights the risk of misinterpreting pHSN as evidence of UVH when considered in isolation. In such cases, findings may be incorrectly interpreted as having vestibular hypofunction rather than a mechanically triggered vestibular disorder. In this patient, extending the examination to include positional testing was critical, as it identified a mechanically triggered etiology that responded immediately to CRM. The HPT was particularly helpful because it demonstrated direction-changing positional nystagmus, which is not typical of vestibular hypofunction but is commonly observed in BPPV. It may also be useful in patients with limited tolerance to provocative maneuvers such as the Dix-Hallpike test. These findings support incorporating positional testing when pHSN is observed without clear lateralizing signs. Based on this case, Fig. 1 summarizes a possible interpretive framework for evaluating pHSN when BPPV is suspected. Resolution of pHSN following CRM may support a transient mechanically triggered rather than fixed neural source of vestibular asymmetry. Persistent findings should prompt further evaluation for hypofunction or central causes.

As a single clinical observation, this case should be interpreted cautiously. Because caloric testing and vHIT were not performed at the initial examination, coexisting vestibular hypofunction cannot be definitively excluded. However, the complete resolution of symptoms, pseudo-spontaneous nystagmus, positional nystagmus, and pHSN after canalith repositioning, together with preserved horizontal vHIT responses at long-term follow-up, supports a transient mechanical contribution rather than a persistent fixed vestibular deficit as the primary explanation.

## Summary

These findings illustrate that spontaneous nystagmus with pHSN should not automatically be attributed to fixed peripheral or central vestibular pathology. In horizontal canal BPPV, transient mechanically triggered vestibular asymmetry may generate findings that resemble vestibular hypofunction. Resolution of pHSN after canalith repositioning supports reassessment after treatment as a useful way to distinguish a transient BPPV-related asymmetry from persistent vestibular dysfunction.

## Supplementary information

Below is the link to the electronic supplementary material.

Supplementary file1 Pseudo-spontaneous nystagmus before treatment (MP4 3339 KB)

Supplementary file2 Post–head-shaking nystagmus before treatment (MP4 8009 KB)

Supplementary file3 Head Pitch Test before treatment (MP4 4585 KB)

Supplementary file4 Supine Head Roll Test before treatment (MP4 10311 KB)

Supplementary file5 Post-treatment resolution (MP4 9306 KB)

Supplementary file6 Horizontal video head impulse testing at 14-month follow-up (PDF 534 KB)

## Data Availability

All data relevant to this case report are included in the article and its supplementary material.

## References

[1] Jacobson GP, Newman CW, Safadi I (1990) Sensitivity and specificity of the head-shaking test for detecting vestibular system abnormalities. Ann Otol Rhinol Laryngol 99(7):539–542 10.1177/000348949009900708 2195962

[2] Hain TC, Fetter M, Zee DS (1987) Head-shaking nystagmus in patients with unilateral peripheral vestibular lesions. Am J Otolaryngol 8(1):36–47 10.1016/s0196-0709(87)80017-0 3578675

[3] Yang T-H, Lee J-H, Oh S-Y, Kang J-J, Kim J-S, Dieterich M (2020) Clinical implications of head‐shaking nystagmus in central and peripheral vestibular disorders: is perverted head‐shaking nystagmus specific for central vestibular pathology? Eur J Neurol 27(7):1296–1303 10.1111/ene.14161 31999861

[4] Lee H, Kim HA (2020) The association of head shaking nystagmus with head-bending and lying-down nystagmus in horizontal canal benign paroxysmal positional vertigo. J Vestib Res 30(2):95–100 10.3233/VES-200696 32310200

[5] Kim M-B, Huh SH, Ban JH (2012) Diversity of head shaking nystagmus in peripheral vestibular disease. Otol Neurotol 33(4):634–639 10.1097/MAO.0b013e31824950c7 22525213

[6] Lopez-Escamez JA, Zapata C, Molina MI, Palma MJ (2007) Dynamics of canal response to head-shaking test in benign paroxysmal positional vertigo. Acta Otolaryngol 127(12):1246–1254 10.1080/00016480701275253 17851954

[7] Lee H, Kim HA (2021) Head-shaking nystagmus in posterior canal benign paroxysmal positional vertigo with canalolithiasis. J Clin Med 10(5) 10.3390/jcm10050916 PMC7956337 33652740

[8] Castellucci A, Malara P, Brandolini C et al (2019) Isolated horizontal canal hypofunction differentiating a canalith jam from an acute peripheral vestibular loss. Am J Otolaryngol 40(2):319–322 10.1016/j.amjoto.2018.12.005 30665622

[9] Babic BB, Jesic SD, Milovanovic JD, Arsovic NA (2014) Unintentional conversion of benign paroxysmal positional vertigo caused by repositioning procedures for canalithiasis: transitional BPPV. Eur Arch Otorhinolaryngol 271(5):967–973. 10.1007/s00405-013-2494-0 23605245

[10] Squires TM, Weidman MS, Hain TC, Stone HA (2004) A mathematical model for the canalithiasis theory of benign paroxysmal positional vertigo. J Biomech 37(8):1137–1146 10.1016/j.jbiomech.2003.12.014 15212918

[11] Curthoys IS, Zee DS, Dumas G, Pastras CJ, Dlugaiczyk J (2025) Skull vibration induced nystagmus, velocity storage and self-stability. Front Neurol 16:1533842. 10.3389/fneur.2025.1533842 PMC11832403 39968451

