# Supplementary material for: Reversible post–head-shaking nystagmus in horizontal canal BPPV as a mechanical mimic of vestibular hypofunction: a case report
Source: Eur Arch Otorhinolaryngol. 2026 Oct 13;283(12):8211–4. doi: 10.1007/s00405-026-10640-y (PMC13768355; doi:10.1007/s00405-026-10640-y)
Supplement: Supplementary file 1 [file 405_2026_10640_MOESM6_ESM.pdf]

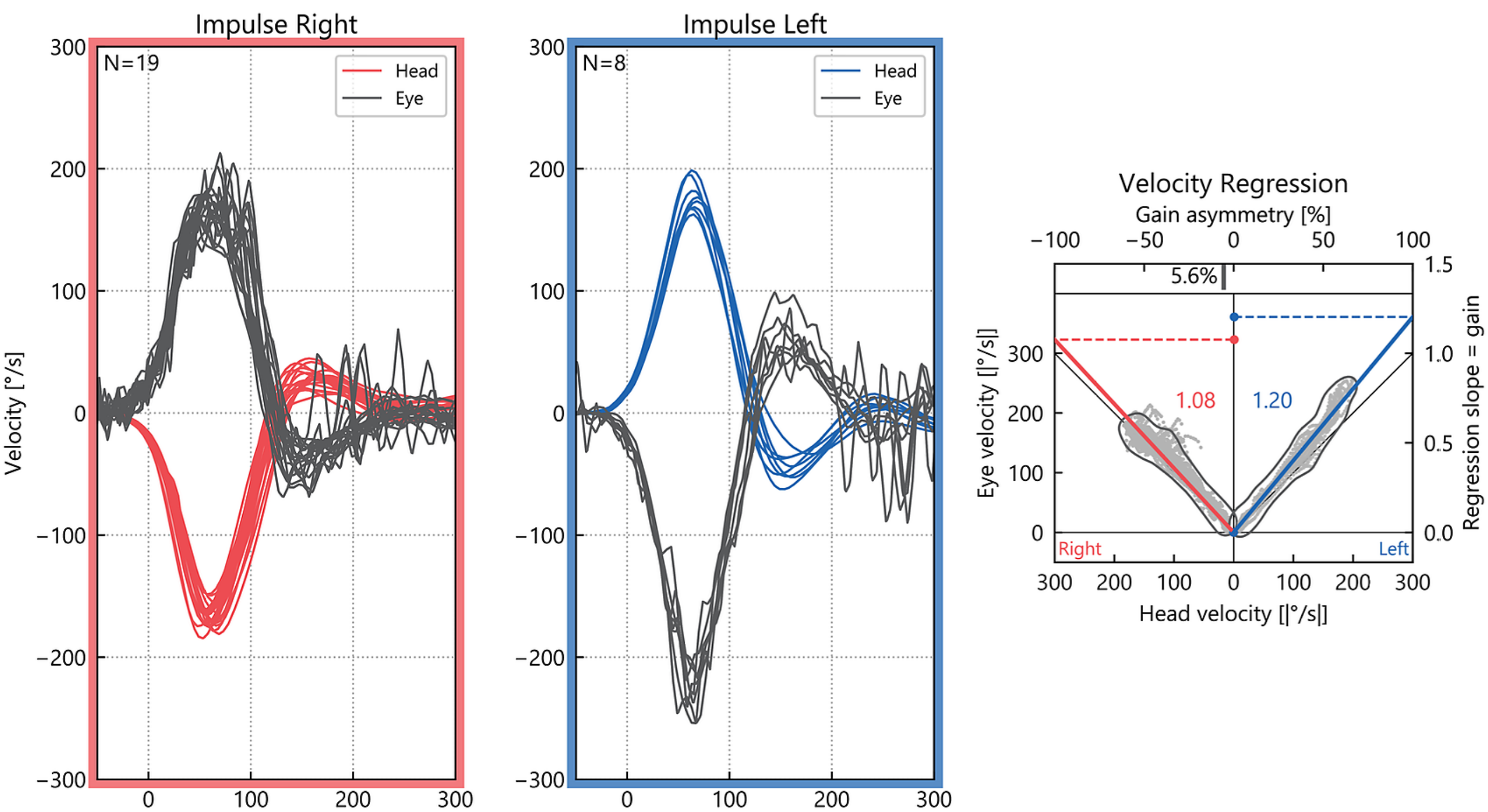

Impulse Right
N=19
Head
Eye
Velocity [°/s]
300
200
100
0
−100
−200
−300
0
100
200
300
Impulse Left
N=8
Head
Eye
Velocity Regression
Gain asymmetry [%]
−100
−50
0
50
100
5.6%
Eye velocity [|°/s|]
Regression slope = gain
1.5
1.0
0.5
0.0
1.08
1.20
Right
Left
Head velocity [|°/s|]
